# Bioprotective Potential of *Pediococcus acidilactici* L1 and *Lactiplantibacillus plantarum* HG1-1 in Harbin Red Sausage Under Vacuum Packaging

**DOI:** 10.3390/foods14244293

**Published:** 2025-12-13

**Authors:** Qiang Wang, Kaida Zhang, Qian Chen, Haotian Liu, Chao Zhang, Qian Liu, Baohua Kong

**Affiliations:** College of Food Science, Northeast Agricultural University, Harbin 150030, China; wangqiangneau@163.com (Q.W.);

**Keywords:** bioprotective potential, bioprotective culture, Harbin red sausage, vacuum packaging, spoilage bacteria

## Abstract

Effective biopreservation strategies are essential to maintain product quality and extend shelf life. However, the low storage temperature (4 °C) of low-temperature meat products limits the growth and activity of most protective cultures, highlighting the need for psychrotrophic strains. This study evaluated the impact of various bioprotective cultures on the bacterial counts, physicochemical quality, flavor profile, and sensory characteristics of the Harbin red sausage under vacuum packaging for 28 days. In comparison with the control (uninoculated) and B2 (commercial *Latilactobacillus sakei* B2) groups, individual and mixed (1:1) inoculations with psychrotrophic *Pediococcus acidilactici* L1 and *Lactiplantibacillus plantarum* HG1-1 significantly inhibited the growth of *Acinetobacter* and *Staphylococcus* (*p* < 0.05), providing the sausage with superior color and texture and delaying lipid oxidation, thereby improving the sausage’s overall acceptability on day 28. The electronic nose analyses indicated that Harbin red sausages inoculated with individual and mixed cultures of *Pe. acidilactici* L1 and *Lac. plantarum* HG1-1 exhibited less development of odor compounds during storage. Overall, both individual and mixed inoculations with *Pe. acidilactici* L1 and *Lac. plantarum* HG1-1 showed superior bioprotective effects on Harbin red sausages under vacuum packaging compared with commercial *Lat. sakei* B2, with the mixed inoculation treatment being the most effective.

## 1. Introduction

Harbin red sausage, as a characteristic meat product in northern China, is favored by consumers for its rich nutrients and unique smoked flavor. Harbin red sausage is a low-temperature meat product, generally heated until the center temperature reaches 74 °C to achieve the cooking requirements [1]. However, this temperature may allow the survival of residual microorganisms, which renders the product more susceptible to spoilage during transportation and retail. With advancements in science and technology, a range of preservation and packaging technologies has been developed to improve the shelf life of food products, including biological and chemical preservatives, high-pressure processing, intense pulse treatment, irradiation, and high-barrier packaging [2]. Among these, metal oxide-coated films such as ZnO-, TiO_2_-, AlOx, or SiOx-based coatings are commonly applied due to their strong barrier properties against oxygen and moisture and their ability to delay microbial spoilage [3]. However, these films can lose functionality when the coating develops cracks, pinholes, or delamination during handling (e.g., vacuum treatment and sterilization) or storage, resulting in increased gas permeability and reduced protective capacity [3]. High-pressure processing can also increase production costs and may cause undesired changes in color and texture; pulsed electric or pulsed light treatments may lead to lipid oxidation or surface discoloration; and irradiation, although effective, often suffers from low consumer acceptance and may induce off-flavors at higher doses [2]. In contrast, the addition of bioprotective bacteria is a reliable, green, healthy, and mild preservation strategy that has received widespread attention [4].

Bioprotective bacteria are live microorganisms that can be added to food products to extend the shelf life of and/or inhibit the growth of harmful microorganisms [5]. The performance of bioprotective cultures is associated with their colonization ability and growth adaptability. Currently, lactic acid bacteria (LAB) have become the focus of bioprotective bacterial screening and application. They possess bioprotective characteristics and have a generally recognized as safe status [6]. LAB can secrete multiple metabolites with bacteriostatic activity, including organic acids, bacteriocins, and cyclic peptides, etc. LAB can also effectively inhibit the growth of spoilage bacteria through their involvement in competitive exclusion and quorum sensing [6]. Effective biopreservation strategies are essential to maintain product quality and extend shelf life. However, the low storage temperature (4 °C) of low-temperature meat products limits the growth and activity of most protective cultures, highlighting the need for psychrotrophic strains. Some LAB possess psychrotrophic traits, enabling them to better adapt to the low-temperature storage environments of meat and meat products. De Andrade Cavalari et al. [7] demonstrated that *Carnobacterium maltaromaticum* (CM-824, CM-827, and CM-289) in ground beef and sliced cooked ham reduced the counts of indigenous spoilage bacteria (*Pseudomonas fluorescens* and *Brochothrix thermosphacta*). Xu et al. [6] reported that *Latilactobacillus sakei* and a mixture of *Staphylococcus carnosus* and *Lat. sakei* controlled microflora and effectively inhibited *B. thermosphacta*, *Pseudomonas*, and *Enterobacteriaceae* in mutton under vacuum packaging.

Regarding the application of bioprotective bacteria, our laboratory has also done some relevant research. Li et al. [8] demonstrated that inoculation with *Staph. xylosus* and *Lactobacillus fermentum* improved the color of raw minced meat and inhibited the growth of spoilage bacteria during the curing process. Wang et al. [5] found that inoculation with *Lat. sakei* B2 and *Lat. curvatus* B48 inhibited the growth of common spoilage bacteria, such as *Ps. fragi*, *Weissella ceti*, and *Serratia proteamaculans*, mitigated odor and color deterioration, and extended the shelf life of the smoked chicken legs under modified atmosphere packaging (MAP) by more than 7 days. Therefore, effective biopreservation strategies are essential to minimize edible meat waste, ensure product quality, and prolong shelf life. Despite the gradual increase in research on bioprotective cultures in recent years, psychrotrophic strains used for the preservation of meat and meat products are still relatively few and need to be developed. In a previous study, we screened *Pediococcus acidilactici* L1 and *Lactiplantibacillus plantarum* HG1-1 from psychrotrophic LAB strains preserved in our laboratory, which exhibited stronger inhibitory activity against *Staph. epidermidis*, *B. thermosphacta*, and *Acinetobacter baumannii* and excellent growth adaptability under low-temperature conditions compared with the commercial bioprotective bacterium *Lat. sakei* B2, suggesting their potential application as a psychrotrophic bioprotective culture in low-temperature meat products [9].

On this basis, this study was conducted to further validate the actual bioprotective ability of the screened *Pe. acidilactici* L1 and *Lac. plantarum* HG1-1 in a complex sausage matrix under commercial vacuum-packaging conditions during refrigerated storage. The aim of this study was to extend our previous findings. The effects of individual and mixed (1:1) inoculation with *Pe. acidilactici* L1 and *Lac. plantarum* HG1-1 on the bacterial counts, physicochemical properties, flavor characteristics, and sensory attributes of Harbin red sausage during storage at 4 °C were investigated. The uninoculated samples were used as the control, and *Lat. sakei* B2 was employed as the commercial control strain. *Pe. acidilactici*, *Lac. plantarum*, and *Lat. sakei* were officially listed in the “Inventory of bacterial species approved for food use” promulgated by the National Health Commission of the People’s Republic of China in 2024.

## 2. Materials and Methods

### 2.1. Bacteria Preparation

The freeze-dried *Lat. sakei* B2 was obtained from Chr. Hansen (Beijing, China). *Pe. acidilactici* L1 was isolated and purified from beef jerky (Harbin, China), and *Lac. plantarum* HG1-1 was isolated and purified from northeastern sauerkraut (Hegang, China). These bacteria were activated twice on Luria—Bertani broth (Hope Bio-Tech, Qingdao, China) at 37 °C. Subsequently, these bacteria were centrifuged at 3000× *g* for 10 min to obtain the bacterial pellet. The bacterial pellet was washed with sterile saline and centrifuged at 3000× *g* for 10 min to obtain the clean bacteria; this step was repeated twice. Finally, the clean bacteria (*Lat. sakei* B2, *Pe. acidilactici* L1, and *Lac. plantarum* HG1-1) were diluted with sterile saline, and approximately 7.0 log CFU/mL of bacterial solution was obtained. The mixed bioprotective bacteria (approximately 7.0 log CFU/mL) were obtained by mixing the bacterial solutions of *Pe. acidilactici* L1 and *Lac. plantarum* HG1-1 in a 1:1 ratio.

### 2.2. Preparation of Harbin Red Sausages

The experiment was conducted in three independent batches on different dates. In each batch, fresh pork and additives were purchased from a local supermarket (Biyoute) in Harbin, China. The Harbin red sausage was formulated with lean pork (10 kg), water (3.32 kg), fat (2.5 kg), starch (0.8 kg), salt (0.3 kg), garlic (132 g), pepper (26 g), monosodium glutamate (26 g), cinnamon (13 g), and sodium nitrite (1 g). According to the method of Wang et al. [10], the Harbin red sausage was prepared by following seven processes: (i) the lean pork and fat were minced separately using a meat grinder; (ii) the lean pork mince and fat were cured separately for 12 h at 4 °C; (iii) the cured lean pork mince and fat were combined with seasonings and subsequently blended with cold water; (iv) the mixture was stuffed into pig intestines to make sausages; (v) the sausages were baked at 65 °C for 60 min; (vi) the sausages were boiled at 85 °C for about 30 min to reach a core temperature of 74 °C; (vii) finally, the sausages were smoked at 60 °C for 3 h to obtain the Harbin red sausages. A total of 450 Harbin red sausages were prepared in three independent batches, with 150 sausages in each batch. The Harbin red sausage is about 18 cm in length, 3.5 cm in diameter, and 110 g in weight.

### 2.3. Inoculation of Bacteria

In each independent batch, the 150 Harbin red sausages were randomly and evenly divided into five groups: (i) without inoculation (control); (ii) inoculation with *Lat. sakei* B2 (B2); (iii) inoculation with *Pe. acidilactici* L1 (L1); (iv) inoculation with *Lac. plantarum* HG1-1 (HG1-1); and (v) inoculation with *Pe. acidilactici* L1 and *Lac. plantarum* HG1-1 (L1 + HG1-1). The 30 samples of each group were immersed in the corresponding bacterial solution (approximately 7.0 log CFU/mL) for 2 min. For each batch, fresh bacterial solution of *Lat. sakei* B2, *Pe. acidilactici* L1, *Lac. plantarum* HG1-1, and the mixed culture of *Pe. acidilactici* L1 and *Lac. plantarum* HG1-1 were independently prepared, ensuring three independent inoculation events. The inoculation amount of the bacteria was set at approximately 5.0 log CFU/g sausage. The actual level of inoculation was determined by the bacterial enumeration on day 0. The samples of the control were treated with sterile saline. In each batch, the raw materials and the inoculation procedures were independently prepared; the three batches represent replicates (n = 3).

### 2.4. Packaging and Storage

In each batch, all Harbin red sausages were individually vacuum-packed. After packaging, all sausages were stored at 4 °C and sampled at 0, 7, 14, 21, and 28 days for analysis. On each sampling day, six Harbin red sausages from each group were used for bacterial enumeration, physicochemical analysis, electronic tongue (E-tongue) analysis, electronic nose (E-nose) analysis, and sensory attribute evaluation.

### 2.5. Bacterial Enumeration

The plate count method was used for bacterial enumeration and to investigate the actual inoculation number and colonization capacity of bioprotective bacteria. For each chop, 25.0 g of Harbin red sausage was homogenized with 225 mL of sterile saline for 2 min. A series of 10-fold dilutions was prepared with sterilized saline. Each dilution was then inoculated onto the corresponding plate count agar, de Man-Rogosa-Sharpe (MRS) agar, mannitol salt agar, and Leeds *Acinetobacter* agar (Hope Bio-Tech, Qingdao, China) for incubation at 37 ± 1 °C for 48 h.

### 2.6. Physicochemical Analysis

The pH and moisture content of Harbin red sausage were determined according to the method of Nie et al. [11]. The color and lipid oxidation of Harbin red sausage were determined according to the methods of Lv et al. [12]. The texture profile analysis (TPA) of Harbin red sausage was conducted using a TA-XT2 plus Texture Analyzer (Stable Micro Systems Ltd., Godalming, UK) equipped with a P50 probe. The sausages were cut into cylinders with a diameter and a height both of 20 mm and a cycle of two compressions was performed with reference to 40% of the height of the sample to determine the texture profiles. The hardness (g), springiness, and cohesiveness of sausages were determined. The water activity of Harbin red sausage was measured using a water activity meter (Aqualab 4TE, Decagon Devices, Inc., Pullman, WA, USA).

### 2.7. Electronic Nose

According to the method of He et al. [13], the E-nose analysis of Harbin red sausages (3.0 g) was performed using a commercial PEN3.5 E-nose (WinMuster Airsense Analytics Inc., Schwerin, Germany). The E-nose was equipped with pattern recognition software and a sensor array unit. It had 10 metal oxide semiconductors, including W1C, W1S, W1W, W2S, W2W, W3C, W3S, W5C, W5S, and W6S, for the specific recognition of different volatile compounds. The sensors are described in Table 1. The measurement duration was 60 s.

### 2.8. Electronic Tongue

According to the method of He et al. [13], the minced Harbin red sausage (30.0 g) was mixed with 150 mL of distilled water. The mixed solution was heated in a 50 °C water bath for 30 min, followed by stirring for 1 min using a tissue masher. Subsequently, the mixed solution was centrifuged for 10 min (5000× *g*, 4 °C), and the supernatant was filtered and used for E-tongue analysis. The TS-5000Z E-tongue instrument (Insent Inc., Atsugi-shi, Japan) was used to analyze the taste attributes of Harbin red sausages. The E-tongue contained five chemical sensors: CA0 (sourness sensor), AE1 (astringency sensor), AAE (umami sensor), CT0 (saltiness sensor), and C00 (bitterness sensor), which are potentiometric sensors with an organic membrane coating with specific sensitivity and selectivity for each sensor.

### 2.9. Sensory Evaluation

Sensory evaluation was conducted in accordance with ISO 11037–2011 [14] standard, as described by Bassey et al. [15]. The sensory panel consisted of 20 fixed members, including faculty, staff, and graduate students. A total of 135 Harbin red sausages (3 batches × 3 sampling days × 5 treatments × 3 sausages) were randomly evaluated in 9 sessions (3 sampling days × 3 batches). In each session, the 20 panelists were trained for approximately 2 h, and 15 samples from all treatment groups (3 samples for each treatment) were randomly coded and evaluated. The samples were cut into 2 mm slices. All panelists participated in each sensory session and evaluated all the samples during the session. Panelists were given sufficient rest between samples to avoid sensory fatigue. The color, hardness, odor, and overall acceptability were evaluated using a 7-point scale. Color: 1 = pale pink; 4 = moderate red; 7 = deep red. Hardness: 1 = soft texture and no springiness; 4 = medium hardness and springiness; 7 = hardness and springiness similar to fresh samples. Odor: 1 = strong spoilage-odor; 4 = off-odor; 7 = no off-odor. Overall acceptability: 1 = low acceptability; 4 = moderate acceptability; 7 = high acceptability.

### 2.10. Statistical Analysis

Three independent experiments were carried out to evaluate the bioprotective potential of cryotolerant bacteria for use in Harbin red sausages. Results were expressed as mean ± standard error. The mixed model analysis was performed using the *nlme* packages in R software (version 4.3.0). The treatment type and storage time were included as fixed effects, and batch (experiment repeated three times) as a random effect. The two-way analyses of variance (ANOVA) and Duncan’s multiple-range test were used for statistical evaluation, with a statistical significance set at *p* < 0.05. The principal component analysis (PCA) was generated using Origin 2021 (version 9.8.0.200).

## 3. Results and Discussion

### 3.1. Bacterial Analysis

#### 3.1.1. Total Viable Counts

**Figure 1:**
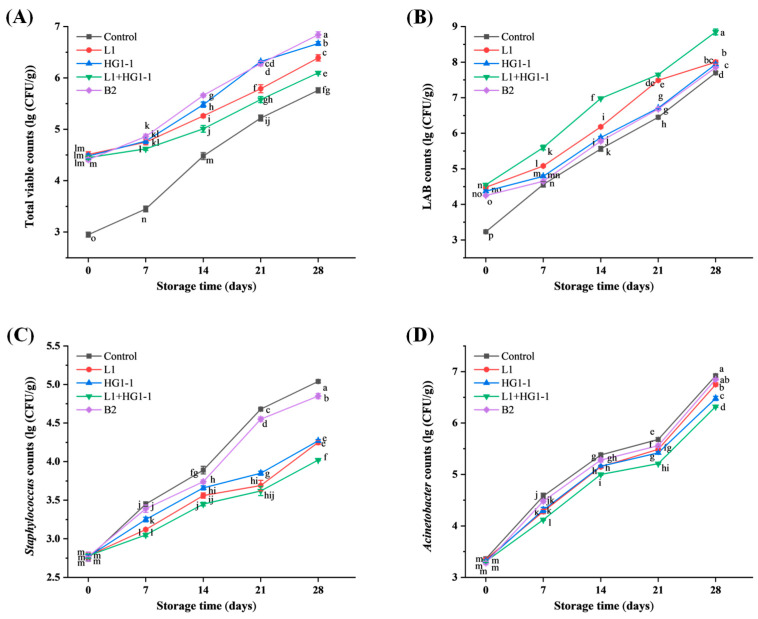
Changes in (**A**) total viable counts, (**B**) lactic acid bacteria counts, (**C**) *Staphylococcus* counts, and (**D**) *Acinetobacter* counts of Harbin red sausages inoculated with different bioprotective cultures during a 28-day storage period at 4 °C. Control: non-inoculation; L1: inoculation with *Pediococcus acidilactici* L1; HG1-1: inoculation with *Lactiplantibacillus plantarum* HG1-1; L1 + HG1-1: mixed (1:1) inoculation with *Pe. acidilactici* L1 and *Lac. plantarum* HG1-1; B2: inoculation with *Latilactobacillus sakei* B2. ^a–p^ Different lowercase letters indicate a significant (*p* < 0.05) difference in all means for viable counts, lactic acid bacteria counts, *Staphylococcus* counts, and *Acinetobacter* counts.

A significant (*p* < 0.05) interaction between bacterial treatment and storage time was observed for the total viable counts (TVC) in Figure 1A. The TVC of L1, HG1-1, L1 + HG1-1, and B2 treatments were approximately 4.50 log CFU/g on day 0, which was significantly (*p* < 0.05) higher than that of the control (2.95 log CFU/g). This may be attributed to the inoculation of LAB. TVC values showed an increasing trend across all treatments, with the control group exhibiting the fastest growth. The B2 treatment demonstrated the highest TVC value (6.85 log CFU/g) at the end of storage, which may be attributed to its higher *Acinetobacter* count (6.84 log CFU/g). The TVC is frequently employed as a standard parameter to evaluate the microbiological quality and shelf life of food products. According to the national standard of the People’s Republic of China (GB, 2726–2016) [16], which considers that the TVCs in meat products below 5 log CFU/g is acceptable. However, TVC cannot differentiate between microbial species. When a bioprotective culture containing large numbers of bacteria is used as a preservative, conventional levels of TVC are no longer suitable for assessing spoilage in meat and meat products [5]. Thus, it is essential to simultaneously evaluate bacterial counts, physicochemical quality, flavor profile, and sensory characteristics.

#### 3.1.2. Lactic Acid Bacteria Counts

A significant (*p* < 0.05) interaction between bacterial treatment and storage time was observed for the LAB counts in Figure 1B. The LAB counts in the control, L1, HG1-1, L1 + HG1-1, and B2 treatments were 3.23, 4.48, 4.37, 4.55, and 4.20 log CFU/g on day 0, respectively, and then showed a significant (*p* < 0.05) increasing trend and reached 7.70, 8.00, 7.94, 8.85, and 7.85 log CFU/g on day 28, respectively. This indicates that *Pe. acidilactici* L1 and *Lac. plantarum* HG1-1 possess excellent colonization ability and growth adaptability during the storage of Harbin red sausage at 4 °C, with the mixed inoculation group exhibiting the highest LAB count. Certainly, the proliferation of bioprotective bacteria may cause a decrease in the pH value of the product.

#### 3.1.3. Staphylococcus Counts

Lv et al. [12] reported that the dominant spoilage bacteria in Harbin red sausage were *Staphylococcus* and *Acinetobacter*. It is effective to assess the bioprotective potential by investigating the inhibitory effect of the bioprotective culture on the dominant spoilage bacteria in the product. A significant (*p* < 0.05) interaction between bacterial treatment and storage time was observed for the *Staphylococcus* counts in Figure 1C. The count of *Staphylococcus* increased from 2.75, 2.78, 2.77, 2.78, and 2.77 log CFU/g on day 0 to 5.04, 4.25, 4.27, 4.02, and 4.85 log CFU/g on day 28 in the control, L1, HG1-1, L1 + HG1-1, and B2 treatments, respectively. Inoculation with *Pe. acidilactici* L1 and *Lac. plantarum* HG1-1 significantly (*p* < 0.05) decreased the count of *Staphylococcus* in the Harbin red sausages compared to the control and B2 treatments, especially the L1 + HG1-1 treatment. The L1 + HG1-1 treatment decreased the *Staphylococcus* count by more than 1.00 and 0.80 log CFU/g at the end of storage compared to the control and B2 treatments. The L1 and HG1-1 treatments also effectively reduced the *Staphylococcus* count by 0.60 and 0.58 log CFU/g, respectively, compared to the B2 treatment.

#### 3.1.4. Acinetobacter Counts

A significant (*p* < 0.05) interaction between bacterial treatment and storage time was observed for the *Acinetobacter* counts in Figure 1D. The count of *Acinetobacter* in each treatment increased significantly (*p* < 0.05). Inoculation with *Pe. acidilactici* L1 and *Lac. plantarum* HG1-1 significantly (*p* < 0.05) decreased the count of *Acinetobacter* in the Harbin red sausages compared to the control treatments on day 28, especially the L1 + HG1-1 treatment, which decreased the count by more than 0.60 log CFU/g. Overall, the L1, HG1-1, and L1 + HG1-1 treatments showed excellent inhibitory ability against both *Acinetobacter* and *Staphylococcus*, which is consistent with the results of our previously published inhibition experiments conducted in plates [9]. Based on the spoilage bacteria predicted by Lv et al. [12] for Harbin red sausage, this study investigated the inhibitory effects of several bioprotective LAB strains against *Staphylococcus* and *Acinetobacter*. However, this approach still has certain limitations and cannot fully characterize the microbial ecosystem and spoilage succession within meat matrices. At present, most studies on bioprotection culture focus on the inhibition of foodborne pathogens such as *Listeria monocytogenes*, *Staph. aureus*, and *Escherichia coli*, as well as common spoilage organisms including *B. thermosphacta*, *Pseudomonas*, and *Enterobacteriaceae*. For example, Stefan and Predescu [17] investigated the efficacy of bioprotective cultures containing *Pe. acidilactici*, a bacteriocin-producing strain known for its antimicrobial activity against *Lis. monocytogenes*. This study demonstrated that *Lis. monocytogenes* counts decreased by 3.2 log CFU/g over 30 days in the batch inoculated with bioprotective cultures, whereas the reduction in the control batch (without bioprotective cultures) was only 1.03 log CFU/g. Casaburi et al. [18] reported that *Lat. curvatus* 54M16 is capable of producing multiple bacteriocins (sakacin X, T, and P) and exhibits antimicrobial activity against *Lis. monocytogenes*, *Bacillus cereus*, and *B. thermosphacta*. In contrast, little is known about the effects of bioprotective cultures on *Staphylococcus* and *Acinetobacter* in meat products. This may be partly attributed to the fact that *Staphylococcus* are often used as starter cultures in fermented meat products rather than considered as spoilage organisms.

### 3.2. pH

**Figure 2:**
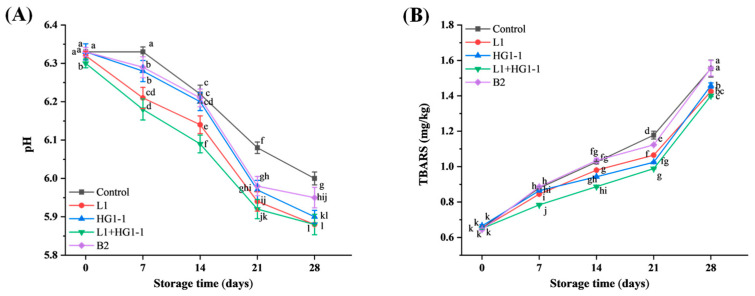
Changes in (**A**) pH and (**B**) thiobarbituric acid reactive substances (TBARS) of Harbin red sausages inoculated with different bioprotective cultures during a 28-day storage period at 4 °C. Control: non-inoculation; L1: inoculation with *Pediococcus acidilactici* L1; HG1-1: inoculation with *Lactiplantibacillus plantarum* HG1-1; L1 + HG1-1: mixed (1:1) inoculation with *Pe. acidilactici* L1 and *Lac. plantarum* HG1-1; B2: inoculation with *Latilactobacillus sakei* B2. ^a–l^ Different lowercase letters indicate a significant (*p* < 0.05) difference in all means for pH and TBARS value.

Although the antimicrobial effect of bioprotective cultures is the main focus, it is equally important to consider their impact on physicochemical quality of the food when evaluating the potential of bioprotective bacteria. A significant (*p* < 0.05) interaction between bacterial treatment and storage time was observed for the pH in Figure 2A. The pH in each treatment showed a significant decrease (*p* < 0.05). The decrease in pH may be due to the production and accumulation of acid by LAB during storage. The pH of each group was about 6.37 on day 0. Subsequently, the pH of the control, L1, HG1-1, L1 + HG1-1, and B2 treatments decreased to 6.01, 5.88, 5.90, 5.88, and 5.95 on day 28. Overall, the inoculation of bioprotective bacteria resulted in a significantly lower pH than the control during storage. The lower pH may also be contributing to the inhibition of spoilage bacteria. Similarly, Wang et al. [5] reported that inoculation with *Lat. sakei* and *Lat. curvatus* significantly decreased the pH of smoked chicken legs, but did not significantly affect the sensory attributes of the product. Trabelsi et al. [19] found that the decrease in pH values resulted from the inoculation of ground beef with the probiotic strain *Lac. plantarum* TN8. In addition, Ben Slima et al. [20] reported that the acidification of probiotic beef sausage samples was due to the production of lactic acid by the LAB.

### 3.3. Lipid Oxidation

Meat and meat products are susceptible to lipid oxidation during storage. Lipid oxidation generally produces volatile organic compounds (VOCs) such as aldehydes, alcohols, acids, and esters, which subsequently affect the odor and acceptability of the product. In general, the presence of oxygen tends to accelerate lipid oxidation. In this study, the Harbin red sausages were stored under vacuum packaging, which greatly reduced the extent of lipid oxidation by limiting oxygen availability. A significant (*p* < 0.05) interaction between bacterial treatment and storage time was observed for the thiobarbituric acid-reactive substances (TBARS) value in Figure 2B. The initial TBARS value for each treatment was about 0.65 mg/kg and then increased significantly (*p* < 0.05). On the one hand, this is partly due to the auto-oxidation of fats during storage; on the other hand, some bacteria produce lipoxygenase, which accelerates the oxidation of unsaturated fatty acids, leading to oxidative rancidity of red sausages [21]. The TBARS values were 1.55, 1.43, 1.46, 1.40, and 1.56 mg/kg on day 28 for the control, L1, HG1-1, L1 + HG1-1, and B2 treatments, respectively. Inoculation with *Pe. acidilactici* L1 and *Lac. plantarum* HG1-1 had an inhibitory effect on lipid oxidation in Harbin red sausage compared to the control and B2 treatments. This may be related to the antibacterial capacity and free radical scavenging activity of *Pe. acidilactici* and *Lac. plantarum* [22]. Similarly, İncili et al. [23] found that the cell-free supernatant of *Pe. acidilactici* significantly inhibited lipid oxidation in frankfurters during storage. Li et al. [24] found that *Lac. plantarum* had strong hydroxyl radical and DPPH free radical scavenging activity, and hydrogen peroxide (H_2_O_2_)-resistant ability. In addition, Trabelsi et al. [19] observed that the addition of *Lac. plantarum* TN8 did not promote lipid oxidation in raw minced beef; on the contrary, it delayed the increase in lipid oxidation compared to the uninoculated control. Katikou et al. [25] found that inoculation with *Lat. curvatus* CECT 904T and *Lat. sakei* CECT 4808 decreased the TBARS value of vacuum-packed beef kept at 4 ± 1 °C for 28 days. In this study, *Pe. acidilactici* L1 and *Lac. plantarum* HG1-1 demonstrated superior inhibitory effects on lipid oxidation compared to *Lat. Sakei* B2.

### 3.4. Moisture Content and Water Activity

Water content and water activity (a_w_) are key determinants of texture, microbial stability, and oxidative reactions in meat products. Variations in moisture and a_w_ typically correlate with changes in hardness, chewiness, and surface color. Therefore, the moisture and a_w_ of products were often analyzed together with texture profile, microbial counts, and lipid oxidation. In Table 2, the interaction between bacterial treatment and storage time on both moisture content and a_w_ was not significant (*p* > 0.05). The moisture content of the Harbin red sausages in each treatment showed a slight decrease with storage. This may be due to a decline in the quality of the Harbin red sausage during storage, resulting in a decrease in water holding capacity, and then the exudation of water under the continuous negative pressure of the vacuum packaging. The a_w_ of Harbin red sausages in all treatments decreased with storage. A similar trend was observed for moisture content. This suggests that the decrease in water activity may be caused by the decrease in moisture content [26].

### 3.5. Textural Properties

**Figure 3:**
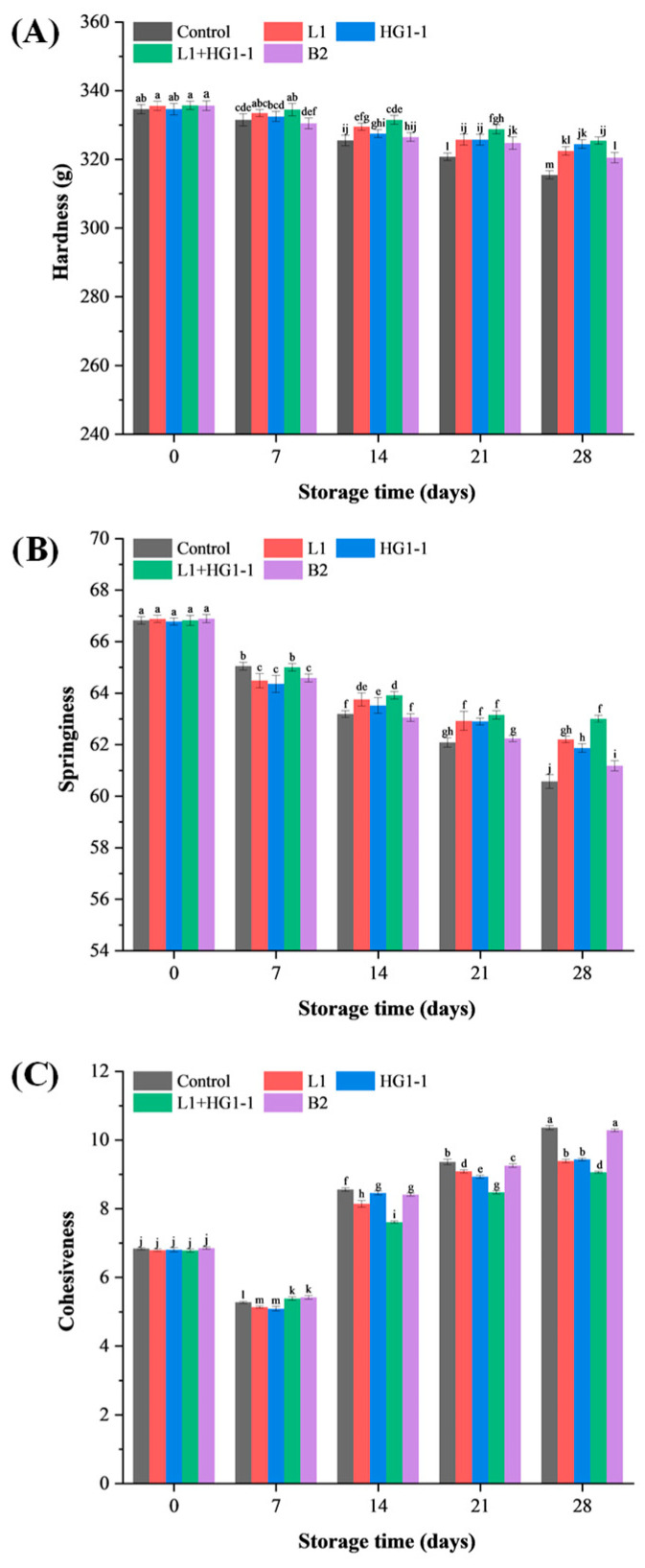
Changes in (**A**) hardness, (**B**) springiness, and (**C**) cohesiveness of Harbin red sausages inoculated with different bioprotective cultures during a 28-day storage period at 4 °C. Control: non-inoculation; L1: inoculation with *Pediococcus acidilactici* L1; HG1-1: inoculation with *Lactiplantibacillus plantarum* HG1-1; L1 + HG1-1: mixed (1:1) inoculation with *Pe. acidilactici* L1 and *Lac. plantarum* HG1-1; B2: inoculation with *Latilactobacillus sakei* B2. ^a–m^ Different lowercase letters indicate a significant (*p* < 0.05) difference in all means for hardness, springiness, and cohesiveness.

The changes in the textural properties of Harbin red sausage may be closely related to moisture content dissipation and quality deterioration. Generally, the dissipation of moisture content may increase the hardness of the product, while the deterioration of quality may decrease the hardness of the product. In Figure 3, a significant interaction between bacterial treatment and storage time was observed for hardness, springiness, and cohesiveness (*p* < 0.05). The L1, HG1-1, and L1 + HG1-1 treatments significantly (*p* < 0.05) delayed the decline in hardness during storage (Figure 3A). On the one hand, it is possible that they decreased the pH through lactic acid production, competitively inhibited the proliferation of spoilage bacteria, and reduced the release of exogenous proteases, thereby counteracting the decrease in hardness [5]. On the other hand, this may be attributed to the fact that a lower pH promotes gel formation. Similarly, Cui et al. [27] reported that inoculation with *Pe*. *acidilactici* and *Lat*. *sakei* in tilapia sausages caused acidification during fermentation, which promoted gel formation and resulted in improved hardness and springiness. Trabelsi et al. [19] reported that the addition of *Lac*. *plantarum* TN8 to minced beef increased hardness. Xu et al. [6] reported that inoculation with *Lat*. *sakei* had no significant main effect on the hardness of lamp during storage (*p* = 0.42). The springiness of all treatments decreased significantly (*p* < 0.05) with storage time, where the springiness of the L1, HG1-1, L1 + HG1-1, and B2 treatments was higher than that of the control, which may be related to the gelatinization effect mentioned above (Figure 3B). Cohesiveness in all treatment groups showed a trend of first decreasing (7 days) and then increasing (Figure 3C). Similarly, Cui et al. [27] reported that the cohesiveness of tilapia sausages inoculated with both *Pe. acidilactici* and *Lat. sakei* first decreased and then increased during storage.

### 3.6. Color

**Figure 4:**
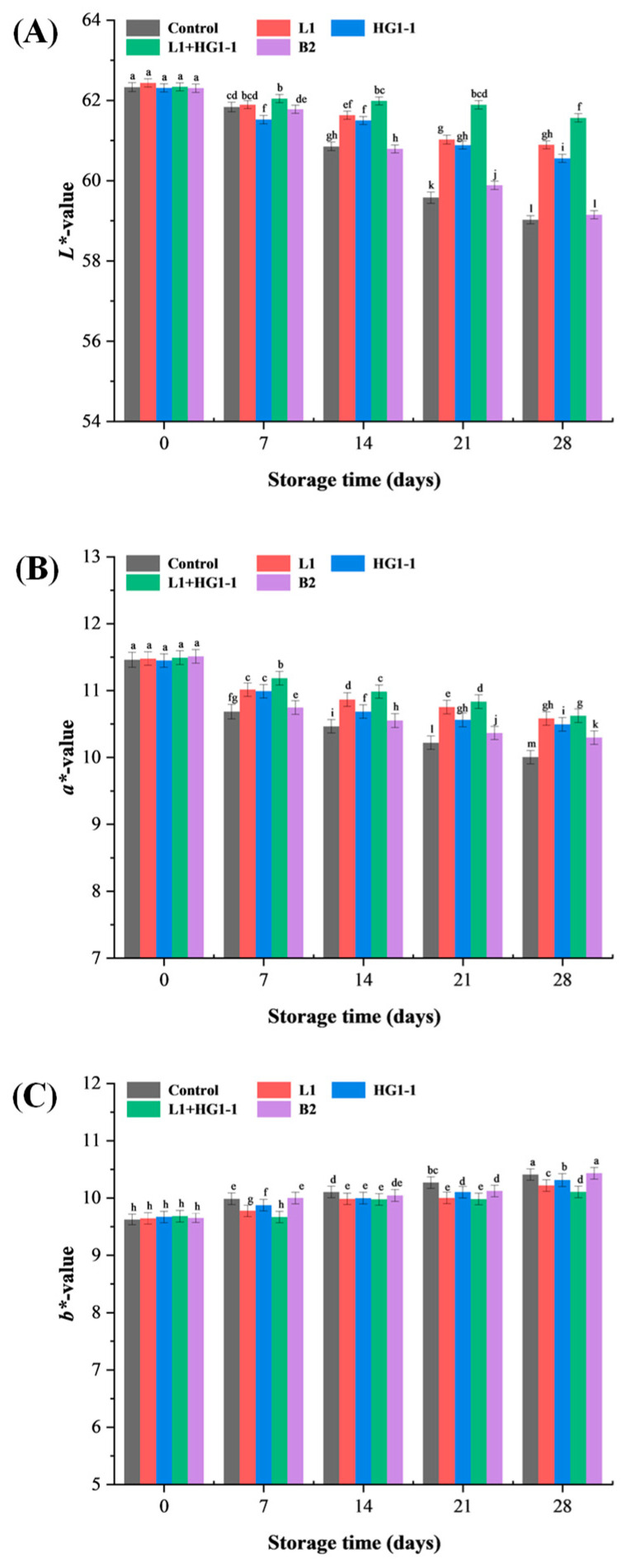
Changes in (**A**) L*, (**B**) a*, and (**C**) b* value of Harbin red sausages inoculated with different bioprotective cultures during a 28-day storage period at 4 °C. Control: non-inoculation; L1: inoculation with *Pediococcus acidilactici* L1; HG1-1: inoculation with *Lactiplantibacillus plantarum* HG1-1; L1 + HG1-1: mixed (1:1) inoculation with *Pe. acidilactici* L1 and *Lac. plantarum* HG1-1; B2: inoculation with *Latilactobacillus sakei* B2. ^a–m^ Different lowercase letters indicate a significant (*p* < 0.05) difference in all means for L*, a*, and b* values.

A significant (*p* < 0.05) interaction between bacterial treatment and storage time was observed for L* values, a* values, browning index, and total color change (ΔE). The L* values of all treatments decreased with increasing storage time (Figure 4A). The L* values of the L1, HG1-1, and L1 + HG1-1 treatments decreased slowly, and, in particular, the L1 + HG1-1 treatment had the most stable L* values, which decreased significantly (*p* < 0.05) after day 21. A significant reduction in a* values was observed across all treatments during storage (*p* < 0.05, Figure 4B), possibly caused by interaction between lipid oxidation products and pigments [12]. Chen et al. [28] also noted that lipid oxidation can contribute to the decline in the surface a* value of roasted duck legs stored under MAP. The a* values of the L1, HG1-1, and L1 + HG1-1 treatments were significantly (*p* < 0.05) higher than those of the control and B2 treatments at the end of the storage, which correlated with the TBARS, implying that the inoculation of *Pe. acidilactici* L1 and *Lac. plantarum* HG1-1 was effective in inhibiting lipid oxidation. The increase in b* values is likely attributable to yellow pigment formation through reactions between lipid oxidation products and amines in the phospholipid head groups or in the proteins [29].

The chroma values of all treatment groups showed minor fluctuations during storage (Table 3). The control and B2 treatments exhibited significant browning and total color changes during storage. Their ΔE values reached 3.70 and 3.49, respectively, on day 28. This may result in lower color and acceptability scores. Overall, inoculation with *Pe. acidilactici* L1 and *Lac. plantarum* HG1-1 did not compromise the color of the Harbin red sausages but instead conferred a bioprotective effect.

### 3.7. Electronic Nose

**Figure 5:**
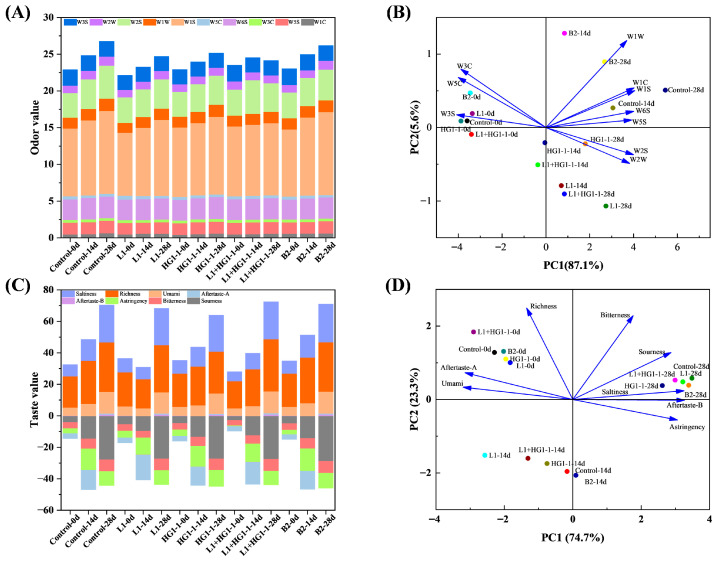
Stacked diagram of electronic nose (**A**), principal component analysis of electronic nose (**B**), stacked diagram of electronic tongue (**C**), and principal component analysis of electronic tongue (**D**) for Harbin red sausages inoculated with different bioprotective cultures. Control: non-inoculation; L1: inoculation with *Pediococcus acidilactici* L1; HG1-1: inoculation with *Lactiplantibacillus plantarum* HG1-1; L1 + HG1-1: mixed (1:1) inoculation with *Pe. acidilactici* L1 and *Lac. plantarum* HG1-1; B2: inoculation with *Latilactobacillus sakei* B2.

E-tongue and E-nose, as bionic devices, can effectively avoid the subjectivity of human response to taste and smell, as well as the variability between individuals, by providing a low-cost, non-invasive, and rapid means of objective taste and odor evaluation of food. In this study, an E-tongue and E-nose were employed to evaluate the taste and smell of Harbin red sausage. However, there are some limitations, as targeted volatile analysis (e.g., HS-SPME/GC-MS) to identify key odor-active compounds was not conducted, nor were taste-active molecules (such as organic acids, amino acids, and nucleotides) quantified. In Figure 5A, the response values of the sensors W3S, W2S, W1S, and W6S were more pronounced, indicating that the samples in all treatments contained elevated levels of long-chain alkanes, alcohols, aldehydes, ketones, alkanes, and hydrides. Similarly, Lv et al. [12] also observed that the contents of alcohols, aldehydes, ketones, and alkanes increased during the storage of Harbin red sausage, particularly ethanol, 3-methyl-1-butanol, 1-octen-3-ol, 2,3-butanediol, hexanal, 1-nonanal, acetoin, and 3-octanone. Most of these VOCs are associated with spoilage odors and are frequently detected in spoiled meat and meat products. The control had the highest total odor response values on days 14 and 28, followed by the B2 treatment. The total odor response values were most stable and lowest at the end of storage for the L1 + HG1-1 treatment. This implies that the inoculation of *Pe. acidilactici* L1 and *Lac. plantarum* HG1-1 did not promote the formation of odor-related substances, but rather inhibited their production to some extent. This may be attributed to the higher odor and overall acceptability scores.

As shown in Figure 5B, two principal components (PC1 and PC2) explained 87.1% and 5.6% of the total variance, respectively. The odor composition of the samples on day 0 of each treatment was relatively similar, all distributed along the negative half-axis of PC1. Notably, L1 + HG1-1-14d and HG1-1-14d were also located on the negative semi-axis of PC1, indicating that their odor compositions had changed less compared to day 0. In addition, L1-14d, L1 + HG1-1-28d, HG1-1-28d, and L1-28d were all in quadrant four, and B2-14d, B2-28d, control-14d, and control-28d were in quadrant one. This implies that Harbin red sausage inoculated with *Pe. acidilactici* L1 and *Lac. plantarum* HG1-1 had a more similar odor composition whether inoculated alone or in a mixture, while Harbin red sausage inoculated with *Lat. sakei* B2 had some similarity in odor to the control.

### 3.8. Electronic Tongue

The sourness, richness, umami, and saltiness of Harbin red sausage of the five groups were enhanced; meanwhile, the remaining taste characteristics fluctuated slightly during the storage period (Figure 5C). This may be related to the organic acids, amino acids, and flavor peptides produced during the storage of Harbin red sausage. In Figure 5D, PC1 and PC2 explained 74.7% and 23.3% of the total variance, respectively. The E-tongue results were distributed into three clusters. On day 0, all treatment groups were distributed in the second quadrant, correlating with richness, aftertaste A (astringent aftertaste), and umami, indicating these taste attributes were prominent at the initial stage. Taste characteristics on day 14 for each treatment group were mainly distributed on the negative half-axis of PC2. At the end of storage (day 28), all treatment groups were located in the first quadrant, correlating with saltiness and sourness, indicating taste alterations in Harbin red sausage during storage. Collectively, storage duration exerted a predominant influence on the taste characteristics of Harbin red sausage.

### 3.9. Sensory Evaluation

Although instrumental and chemical analyses provide essential objective insights into the application of bioprotective bacteria as preservation agents, sensory evaluation is crucial since it directly determines consumer acceptance. A significant (*p* < 0.05) interaction between bacterial treatment and storage time was observed for color, hardness, odor, and overall acceptability in Table 4. On day 0, there were no significant (*p* > 0.05) differences in sensory scores between the control and inoculated groups, indicating that the inoculation of bioprotective cultures did not affect the initial sensory properties of Harbin red sausages. With prolonged storage time, all treatment groups exhibited significant decreases (*p* < 0.05) in internal color, odor, hardness, and overall acceptability. On day 28, the internal color scores of the L1, HG1-1, L1 + HG1-1, and B2 treatments were significantly higher (*p* < 0.05) than those of the control group (2.98). These results demonstrate the color preservation effects of the bioprotective cultures. The L1, HG1-1, and L1 + HG1-1 treatments maintained superior hardness at storage termination, showing significant differences (*p* < 0.05) compared to the control treatments, which aligned with the texture profile analysis outcomes. The L1, HG1-1, and L1 + HG1-1 treatments demonstrated higher odor scores at storage termination. The results of overall acceptability revealed that the L1 and L1 + HG1-1 groups demonstrated the highest scores (3.86 and 3.95, respectively) at the end of storage, followed by the HG1-1 and B2 groups with values of 3.80 and 3.64. The control group exhibited the lowest score of 3.02. This might be attributed to the superior antibacterial capacity observed in L1 and L1 + HG1-1 treatments. Based on the overall acceptability scores, the bioprotective culture treatments delayed spoilage by approximately 14 days compared to the control treatment. In conclusion, the inoculation of bioprotective cultures *Pe. acidilactici* L1, *Lac. plantarum* HG1-1, and *Lat. sakei* B2 effectively extended the shelf life of Harbin red sausages, with *Pe. acidilactici* L1 and *Lac. plantarum* HG1-1 demonstrating superior bioprotective effects, particularly under mixed inoculation, where the most pronounced protection was observed.

### 3.10. General Discussion

The present study comprehensively evaluated the bioprotective potential of *Pe. acidilactici* L1 and *Lac. plantarum* HG1-1 in Harbin red sausages under vacuum packaging at 4 °C, integrating microbiological, physicochemical, and sensory assessments. The inoculation of these strains significantly inhibited the growth of *Staphylococcus* and *Acinetobacter*, which is consistent with their previously observed antimicrobial activity in vitro [9]. The mixed inoculation (L1 + HG1-1) exhibited the strongest inhibition, highlighting a synergistic effect in the co-culture system. The inhibitory effects on *Staphylococcus* and *Acinetobacter* may be associated with the acid production of *Pe. acidilactici* L1 and *Lac. plantarum* HG1-1. The lower pH may reinforce microbial inhibition. Correspondingly, hardness and springiness were better preserved in the L1, HG1-1, and L1 + HG1-1 groups, which may be attributed to the antimicrobial effects of *Pe. acidilactici* L1 and *Lac. plantarum* HG1-1, as well as the potential gelation effects [27].

Furthermore, the inoculation of *Pe. acidilactici* L1 and *Lac. plantarum* HG1-1 effectively delayed lipid oxidation, as indicated by lower TBARS values, which was likely associated with both free radical scavenging activity and microbial inhibition [22]. These biochemical changes were reflected in color stability, where L* and a* values were maintained more consistently, and browning and total color changes (ΔE) were minimized, supporting higher sensory scores.

The E-nose analyses further demonstrated that inoculated sausages exhibited less development of off-odor compounds during storage. This suggests that the bioprotective cultures contributed to the preservation of organoleptic quality. Overall, these results demonstrate that *Pe. acidilactici* L1 and *Lac. plantarum* HG1-1 extend the shelf life through multifactorial mechanisms, including microbial inhibition, acidification, antioxidant activity, and maintenance of texture and color.

## 4. Conclusions

This study demonstrated the potential of *Pe. acidilactici* L1 and *Lac. plantarum* HG1-1 as bioprotective cultures in Harbin red sausages stored in vacuum packaging at 4 °C. Compared with the control (uninoculated) and B2 treatments, the L1, HG1-1, and L1 + HG1-1 treatments significantly (*p* < 0.05) inhibited the growth of *Acinetobacter* and *Staphylococcus*, maintained better color and texture characteristics, delayed lipid oxidation, and delayed flavor deterioration, resulting in higher overall acceptability scores at the end of storage. The B2 group also showed a certain bioprotective effect compared with the control group, but its overall effect was relatively weaker. In summary, both the individual and mixed inoculations of the *Pe. acidilactici* L1 and *Lac. plantarum* HG1-1 provided superior bioprotective effects on Harbin red sausages under vacuum packaging compared with the commercial strain *Lat. sakei* B2. Based on the overall acceptability scores, the bioprotective culture treatments delayed spoilage by approximately 14 days compared to the control treatment. In conclusion, the inoculation of bioprotective cultures *Pe. acidilactici* L1, *Lac. plantarum* HG1-1, and *Lat. sakei* B2 effectively delayed the spoilage process of Harbin red sausages, with *Pe. acidilactici* L1 and *Lac. plantarum* HG1-1 demonstrating superior bioprotective effects, particularly under mixed inoculation. Based on these results, it can be deduced that *Pe. acidilactici* L1 and *Lac. plantarum* HG1-1 are capable of adapting to the complex matrices and refrigerated conditions of most meat products as bioprotective bacteria. This study validates the bioprotective capacity of *Pe. acidilactici* L1 and *Lac. plantarum* HG1-1, provides a useful reference for the selection of psychrotrophic bioprotective cultures, and offers practical insights for the implementation of biopreservation strategies in low-temperature meat products.

## Figures and Tables

**Table 1:** Electronic nose (E-nose) sensors and their representative materials.

Sensor Name	Representative Material Species	Representative Material
W1C	Aromatic compounds	Sensitive to aromatic constituents, benzene
W5S	Broad range	Sensitive to nitrogen oxides
W3C	Aromatic	Sensitive to aroma, ammonia
W6S	Hydrogen	Sensitive to hydrides
W5C	Arom-aliph	Sensitive to short-chain alkane aromatic component
W1S	Broad-methane	Sensitive to methyl
W1W	Sulphur-organic	Sensitive to sulfides
W2S	Broad-alcohol	Sensitive to alcohols, aldehydes and ketones
W2W	Sulph-chlor	Sensitive to organic sulfides
W3S	Methane-aliph	Sensitive to long-chain alkanes

**Table 2:** Changes in moisture content and water activity (a_w_) of Harbin red sausages inoculated with different bioprotective cultures during a 28-day storage period at 4 °C.

	Storage Time	Treatment				
		Control	L1	HG1-1	L1 + HG1-1	B2
Moisture content (%)	0	58.56 ± 0.01 ^b^	58.55 ± 0.01 ^b^	58.55 ± 0.01 ^b^	58.52 ± 0.01 ^bc^	58.49 ± 0.48 ^c^
	7	58.40 ± 0.01 ^d^	58.48 ± 0.47 ^c^	58.49 ± 0.01 ^c^	58.50 ± 0.01 ^c^	58.41 ± 0.01 ^d^
	14	58.89 ± 0.03 ^a^	57.98 ± 0.01 ^e^	57.97 ± 0.24 ^e^	57.99 ± 0.07 ^e^	57.88 ± 0.02 ^f^
	21	57.85 ± 0.01 ^f^	57.86 ± 0.01 ^f^	57.85 ± 0.02 ^f^	57.86 ± 0.01 ^f^	57.84 ± 0.02 ^fg^
	28	57.80 ± 0.02 ^g^	57.82 ± 0.01 ^fg^	57.81 ± 0.01 ^fg^	57.83 ± 0.01 ^fg^	57.81 ± 0.01 ^fg^
Water activity (a_w_)	0	0.99 ± 0.01 ^a^	0.98 ± 0.01 ^a^	0.99 ± 0.01 ^a^	0.98 ± 0.01 ^a^	0.98 ± 0.01 ^a^
	7	0.97 ± 0.01 ^ab^	0.97 ± 0.01 ^ab^	0.98 ± 0.01 ^a^	0.97 ± 0.01 ^ab^	0.97 ± 0.01 ^ab^
	14	0.96 ± 0.01 ^ab^	0.96 ± 0.01 ^ab^	0.97 ± 0.01 ^ab^	0.97 ± 0.01 ^ab^	0.97 ± 0.01 ^ab^
	21	0.95 ± 0.01 ^ab^	0.96 ± 0.02 ^ab^	0.96 ± 0.01 ^ab^	0.96 ± 0.01 ^ab^	0.96 ± 0.01 ^ab^
	28	0.94 ± 0.01 ^b^	0.95 ± 0.01 ^ab^	0.95 ± 0.01 ^ab^	0.95 ± 0.01 ^ab^	0.93 ± 0.02 ^b^

Control: non-inoculation; L1: inoculation with *Pediococcus acidilactici* L1; HG1-1: inoculation with *Lactiplantibacillus plantarum* HG1-1; L1 + HG1-1: mixed (1:1) inoculation with *Pe. acidilactici* L1 and *Lac. plantarum* HG1-1; B2: inoculation with *Latilactobacillus sakei* B2. ^a–g^ Different lowercase letters indicate a significant (*p* < 0.05) difference in all means for moisture content and a_w_.

**Table 3:** Changes in browning index, chroma, and total color of Harbin red sausages inoculated with different bioprotective cultures during a 28-day storage period at 4 °C.

	Storage Time (Days)	Treatment				
		Control	L1	HG1-1	L1 + HG1-1	B2
Browning index	0	29.73 ± 0.07 ^j^	29.73 ± 0.03 ^j^	29.81 ± 0.11 ^ij^	29.87 ± 0.16 ^hi^	29.85 ± 0.15 ^hi^
	7	29.80 ± 0.11 ^ij^	29.73 ± 0.09 ^j^	30.09 ± 0.08 ^g^	29.64 ± 0.07 ^k^	29.92 ± 0.11 ^ghi^
	14	30.28 ± 0.06 ^fg^	30.10 ± 0.13 ^g^	29.99 ± 0.02 ^ghi^	30.04 ± 0.08 ^gh^	30.30 ± 0.05 ^fg^
	21	31.01 ± 0.14 ^c^	30.32 ± 0.07 ^fg^	30.37 ± 0.04 ^f^	29.93 ± 0.07 ^ghi^	30.72 ± 0.09 ^de^
	28	31.35 ± 0.17 ^b^	30.62 ± 0.04 ^e^	30.89 ± 0.09 ^cd^	30.10 ± 0.12 ^g^	31.68 ± 0.14 ^a^
Chroma	0	14.96 ± 0.09 ^a^	14.99 ± 0.08 ^a^	14.98 ± 0.11 ^a^	15.02 ± 0.07 ^a^	15.02 ± 0.10 ^a^
	7	14.62 ± 0.17 ^cde^	14.72 ± 0.11 ^abc^	14.77 ± 0.16 ^ab^	14.78 ± 0.09 ^ab^	14.68 ± 0.08 ^bcd^
	14	14.54 ± 0.13 ^cde^	14.75 ± 0.08 ^abc^	14.63 ± 0.07 ^cde^	14.84 ± 0.16 ^ab^	14.57 ± 0.12 ^cde^
	21	14.48 ± 0.12 ^de^	14.68 ± 0.08 ^bcd^	14.61 ± 0.04 ^cde^	14.73 ± 0.13 ^abc^	14.49 ± 0.09 ^de^
	28	14.43 ± 0.08 ^e^	14.71 ± 0.15 ^bcd^	14.71 ± 0.15 ^bcd^	14.66 ± 0.06 ^bcd^	14.65 ± 0.13 ^bcd^
Total color change (ΔE)	0	-	0.11 ± 0.03 ^l^	0.05 ± 0.03 ^l^	0.06 ± 0.02 ^l^	0.06 ± 0.03 ^l^
	7	0.99 ± 0.13 ^i^	0.65 ± 0.09 ^j^	0.97 ± 0.03 ^i^	0.40 ± 0.05 ^k^	0.98 ± 0.05 ^i^
	14	1.85 ± 0.05 ^f^	0.99 ± 0.10 ^i^	1.20 ± 0.07 ^h^	0.69 ± 0.07 ^j^	1.84 ± 0.09 ^f^
	21	3.09 ± 0.06 ^c^	1.54 ± 0.08 ^g^	1.77 ± 0.06 ^f^	0.85 ± 0.13 ^ij^	2.73 ± 0.13 ^d^
	28	3.70 ± 0.14 ^a^	1.79 ± 0.09 ^f^	2.14 ± 0.16 ^e^	1.23 ± 0.05 ^h^	3.49 ± 0.11 ^b^

Control: non-inoculation; L1: inoculation with *Pediococcus acidilactici* L1; HG1-1: inoculation with *Lactiplantibacillus plantarum* HG1-1; L1 + HG1-1: mixed (1:1) inoculation with *Pe. acidilactici* L1 and *Lac. plantarum* HG1-1; B2: inoculation with *Latilactobacillus sakei* B2. ^a–l^ Different lowercase letters indicate a significant (*p* < 0.05) difference in all means for browning index, chroma, and total color change.

**Table 4:** Changes in sensory scores of Harbin red sausages inoculated with different bioprotective cultures during a 28-day storage period at 4 °C.

Sensory Attribute	Storage Time (Days)	Treatment				
		Control	L1	HG1-1	L1 + HG1-1	B2
Color	0	6.43 ± 0.24 ^a^	6.41 ± 0.25 ^a^	6.40 ± 0.26 ^a^	6.44 ± 0.27 ^a^	6.41 ± 0.28 ^a^
	14	3.80 ± 0.31 ^c^	3.90 ± 0.21 ^bc^	3.92 ± 0.23 ^bc^	4.08 ± 0.27 ^b^	3.82 ± 0.21 ^c^
	28	2.98 ± 0.32 ^f^	3.56 ± 0.28 ^de^	3.50 ± 0.31 ^de^	3.66 ± 0.28 ^d^	3.41 ± 0.31 ^e^
Hardness	0	6.21 ± 0.21 ^a^	6.24 ± 0.32 ^a^	6.23 ± 0.24 ^a^	6.25 ± 0.28 ^a^	6.24 ± 0.24 ^a^
	14	5.75 ± 0.31 ^cd^	5.92 ± 0.27 ^b^	5.95 ± 0.25 ^b^	5.92 ± 0.26 ^b^	5.81 ± 0.27 ^bc^
	28	4.95 ± 0.33 ^g^	5.58 ± 0.35 ^e^	5.56 ± 0.34 ^e^	5.68 ± 0.32 ^de^	5.27 ± 0.28 ^f^
Odor	0	6.49 ± 0.22 ^a^	6.51 ± 0.21 ^a^	6.50 ± 0.29 ^a^	6.48 ± 0.25 ^a^	6.49 ± 0.25 ^a^
	14	3.86 ± 0.27 ^c^	4.08 ± 0.21 ^b^	4.05 ± 0.28 ^bc^	4.10 ± 0.27 ^b^	3.90 ± 0.24 ^c^
	28	3.06 ± 0.22 ^f^	3.22 ± 0.31 ^de^	3.28 ± 0.21 ^de^	3.36 ± 0.25 ^d^	3.16 ± 0.24 ^ef^
Overall acceptability	0	6.53 ± 0.29 ^a^	6.55 ± 0.27 ^a^	6.55 ± 0.26 ^a^	6.54 ± 0.25 ^a^	6.52 ± 0.31 ^a^
	14	3.78 ± 0.31 ^de^	4.10 ± 0.21 ^bc^	4.02 ± 0.31 ^bcd^	4.20 ± 0.31 ^b^	4.06 ± 0.28 ^bc^
	28	3.02 ± 0.23 ^f^	3.86 ± 0.32 ^d^	3.80 ± 0.31 ^d^	3.95 ± 0.32 ^cd^	3.64 ± 0.34 ^e^

Control: non-inoculation; L1: inoculation with *Pediococcus acidilactici* L1; HG1-1: inoculation with *Lactiplantibacillus plantarum* HG1-1; L1 + HG1-1: mixed (1:1) inoculation with *Pe. acidilactici* L1 and *Lac. plantarum* HG1-1; B2: inoculation with *Latilactobacillus sakei* B2. ^a–g^ Different lowercase letters indicate a significant (*p* < 0.05) difference in all means for color, hardness, odor, and overall acceptability.

## Data Availability

The original contributions presented in the study are included in the article. Further inquiries can be directed to the corresponding author.
